# Evaluating Major Electrode Types for Idle Biological Signal Measurements for Modern Medical Technology

**DOI:** 10.3390/bioengineering3030020

**Published:** 2016-08-24

**Authors:** Anas Albulbul

**Affiliations:** Department of Research and Development, Global Innovative Medical Technologies (GIMT), Ottawa, ON K1G 5L1, Canada; a-albulbul@gimt.com; Tel.: +1-613-663-8611

**Keywords:** biological signals, electrodes, electrode-skin impedance, noise

## Abstract

Biological signals such as electrocardiogram (ECG) and electromyography (EMG) that can be measured at home can reveal vital information about the patient’s health. In today modern technology, the measured ECG or EMG signals at home can be monitored by medical staff from long distance through the use of internet. Biopotential electrodes are crucial in monitoring ECG, EMG, etc., signals. Applying the right type of electrode that lasts for a long time and assists in recording high signal quality is desirable in medical devices industry. Three types of electrodes (Silver/Silver Chloride (Ag/AgCl) electrodes, Orbital electrodes and Stainless steel electrodes) were tested to identify the most appropriate one for recording biological signals. The evaluation was based on determining the electrode circuit model components and having high capacitance value or high capacitor value of electrode circuit model (C_d_) and low electrode-skin impedance value or low resistor value of electrode circuit model (R_d_). The results revealed that Ag/AgCl is the best type of electrodes, followed by Orbital electrodes. Stainless steel electrodes had performed poorly. However, Orbital electrodes material can last longer than Ag/AgCl and hence perform similar to Ag/AgCl electrodes, which can be idle for monitoring biological signals at home without the need for medical staff to replace the electrodes in a short period of time.

## 1. Introduction

The implications of smart devices at home and the development of medical technologies have improved the healthcare home devices. Monitoring the patient’s health condition at home has become crucial in the current modern world.

Biological signals, such as electrocardiogram (ECG), electromyogram (EMG), and electroencephalogram (EEG), are rich in medical information. Biopotential electrodes are designed to assist in measuring and recording biological signals. Biopotential electrodes have the ability to transduce bioelectric activity within the body (ionic current) into electrical current that can be measured and recorded [1,2]. The performance of non-invasive electrodes in detecting biological signals is highly dependent on electrode-skin impedance [3,4].

High electrode-skin impedance would result in poor biological signal quality, low signal amplitude and low signal to noise ratio [2,5]. Selecting the proper type of electrodes that can result in having low electrode-skin impedance and can last longer for recording is important for bio-signal measurements.

The main problem of conducting bio-signal measurements at home is the choice of an appropriate bioptential electrode that can last long time and need minimal preparation work for recording bio-signal measurements. 

The main objective of this research paper is to compare the performance of the most common non-invasive biopotential electrodes to benefit the medical industry in choosing the most appropriate type of electrodes for clinical measurements at home. 

### 1.1. Biopotential Electrodes 

Ideal non-polarizable electrodes permit the charges to pass through the electrode-skin interface without hindrance [5]. In non-polarizable electrodes, reduction/oxidation reactions occur at the electrode-skin interface, exchanging charge carriers from ions to electrons and vice versa [5,6,7,8]. These reactions are electrochemically reversible in non-polarizable electrodes [5]. The electrolyte gel is used with non-polarizable electrodes to facilitate the electrochemical reactions and to reduce electrode-skin interface impedance [4,5,9]. 

Stainless steel electrodes are classified as polarizable electrodes [1]. They are one of the most common polarizable electrodes used in modern wireless sensor technologies for monitoring biological signals (e.g., chairs, shirts) [10,11]. 

Ag/AgCl electrodes are classified as non-polarizable electrodes and considered as the universal electrodes in clinical measurements (e.g., ECG, EMG and EEG) [1]. They are associated with low electrode-skin impedance, low noise and low motion artifact [12].

### 1.2. Electrode-Skin Impedance 

Electrode-skin impedance plays a major role in biological signal quality. High electrode-skin impedance influences negatively biological signal quality since it is associated with low signal-to-noise ratio [13]. High electrode-skin impedance causes poor detection of biopotentials at the electrodes sites because it forms a strong barrier for the biopotentials to cross it [1]. High electrode-skin impedance could be linked with low mobility of ions across the highly resistant skin layer (stratum corneum) that is in contact with electrodes and low electron/ion exchange at electrodes sites [2,5]. Thus, that could cause weak conductivity between the electrodes and the skin and would reduce the biological signal amplitude (low signal to noise ratio). A mismatch in impedance between the electrodes at the skin surface during recording a biological signal would reduce the common mode rejection ratio of the recording system, increase common mode interference (e.g., power line noise) and decrease the signal-to-noise ratio [5]. 

Electrode-skin impedance varies from one person to another and from one part of the body to another. For example, when Rosell et al. measured the electrode-skin impedance at different parts of the body for ten subjects using Ag/AgCl electrodes, they found a high electrode-skin impedance of around 1 MΩ at 1 Hz at the leg site, and around 100 kΩ at the forehead site [4].

Non-polarizable electrodes are likely to have lower electrode-skin impedances in comparison to polarizable electrodes [14,15].

### 1.3. Properties of Ag/AgCl Electrodes 

Surface Ag/AgCl electrodes are the most common and favoured electrodes in clinical measurements for recording biological signals such as ECG, EMG and EEG [16]. One of the main advantages of using Ag/AgCl electrodes is the low noise level it generates during biological signals recording [16]. Ag/AgCl electrodes generate lower electrode-skin interface impedance and lower electrode-skin interface impedance value than stainless steel electrodes [16,17,18]. They are also considered as non-polarizable electrodes; the non-polarizable nature of Ag/AgCl electrodes allows the charges to cross the electrode-electrolyte interface unlike stainless steel electrodes [7,17,18,19]. 

### 1.4. Properties of Orbital Electrodes 

Dry polarizable Orbital electrodes are made to last longer than the common clinical wet electrodes such as Ag/AgCl [20,21]. An orbital electrode’s coat is made of a mixture of metals: silver/silver chloride, aluminum, gold/gold chloride, nickel and titanium [21]. The Orbital Research Inc. stated that the main advantages of applying Orbital electrodes are the elimination for the need of skin preparation and for an electrolyte gel application during the biological signal recording period [21]. 

**Figure 1:**
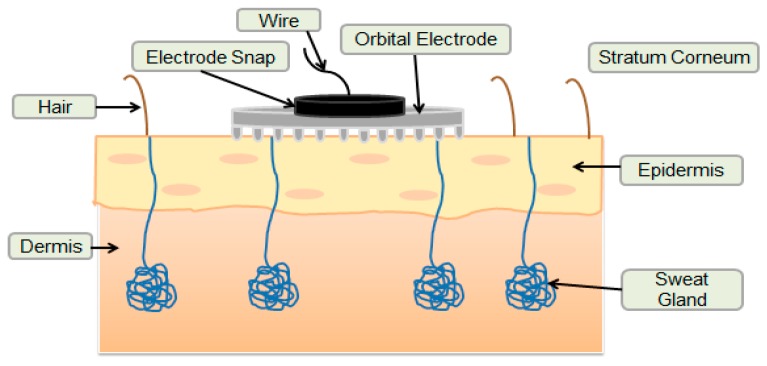
Orbital electrode’s penetration into the skin layers during bio-signal recording.

The shape of the Orbital electrode makes it more in contact with the skin than is the case with regular flat stainless steel or surface Ag/AgCl electrodes. This is due to the presence of pins (spikes) with a height of approximately 150 μm, which allow the Orbital electrode to penetrate deeper into the stratum corneum layer that dominates the skin’s surface and thus facilitates the pathways for biopotential through the skin to the Orbital electrode (Figure 1) [20,21]. Stratum corneum has a high resistance to biopotentials and to electrical current due to the presence of dead skin cells [2,16]. The application of Orbital electrode can overcome this problem by the presence of pins [20,21].

### 1.5. Properties of Stainless Steel Electrodes 

Dry electrodes such as stainless steel electrodes are classified as polarizable electrodes [7,22]. The research performed by Ragheb and Geddes was based on measuring the electrode-electrolyte interface impedance at frequencies range from 1 Hz to 1 MHz [7]. The results showed that stainless steel electrode had high impedance in a range of 30–75 kΩ at low frequency range 100 Hz [7]. Stainless steel electrodes would generate higher electrode-skin interface impedance than the other types of electrodes [7]. Furthermore, polarizable electrodes such as surface stainless steel electrodes can be reused due to their resistance to corrosion [1].

### 1.6. Measuring the Electrode-Skin Impedance 

An equivalent circuit model can be used to better understand the interactions between a surface electrode and the skin. Warburg was known to be the first to propose an equivalent electrode-electrolyte interface circuit model [23]. Feates et al. had identified the components of the equivalent electrode circuit model by analyzing the conductivity nature of biological tissues [24]. Their work helped in estimating the values of capacitors and resistors in the electrode-skin model. In addition, their study provided more details on the effect of skin capacitance, impedance and electrolyte gel or sweat on the electrode-skin impedance.

## 2. Materials and Methods 

A bioimpedance measurement system is used to measure the electrode-skin impedance in response to different frequencies and to an applied alternating electrical current in accordance with the safety standards. 

### 2.1. Measurement Devices 

The bioimpedance measurement system used in the study consists of a personal computer (PC) (Dell 390, Processor 3.0 GHz, Pentium 2, Win XP), frequency response analyzer (FRA) (Model # 1255B, Solartron Analytical, Farnborough, UK) and an impedance interface device (Model # 1294A, Solartron Analytical, Farnborough, UK). 

Impedance was measured from 1 Hz to 1 MHz (10 points per decade), averaging 20 cycles per frequency, with applying an alternating electrical current of 100 μA root mean square supply current. The applied alternating electrical current 100 μA is in accordance with the safety standards. A value of 100 μA is a low AC current value that may not harm the human body [5]. 

### 2.2. Measurements

Each impedance measurement took approximately 6 min to complete. Two electrodes from the same type were placed on the ventral side of the right forearm, spaced 7 cm apart, with the distal electrode approximately 11 cm from the wrist. The measurements were done without performing skin preparation at the electrodes sites and performed immediately after placing the electrodes. Five human subjects were participated in the study (Table 1). This study was reviewed and approved by Carleton University Research Ethics Committee, approval # 12-0350 and it was carried out following the rules of the Declaration of Helsinki of 1975. All subjects gave their informed consent for inclusion before they participated in the study.

### 2.3. Electrodes 

**Figure 2:**
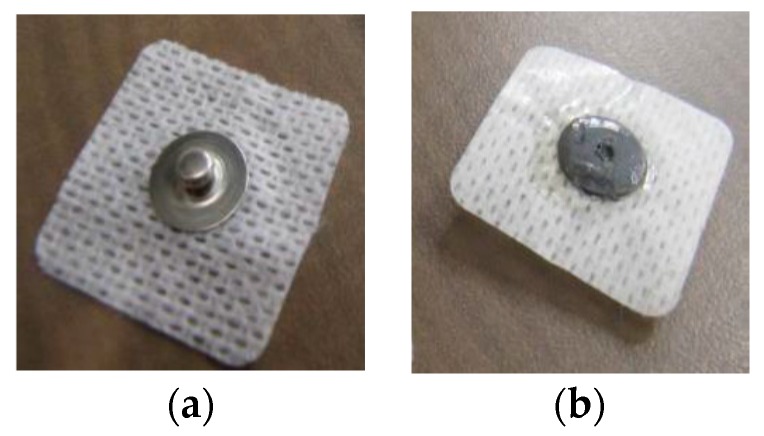
(**a**) Ag/AgCl electrode (electrode’s snap side); (**b**) Ag/AgCl electrode (electrode’s skin side).

**Figure 3:**
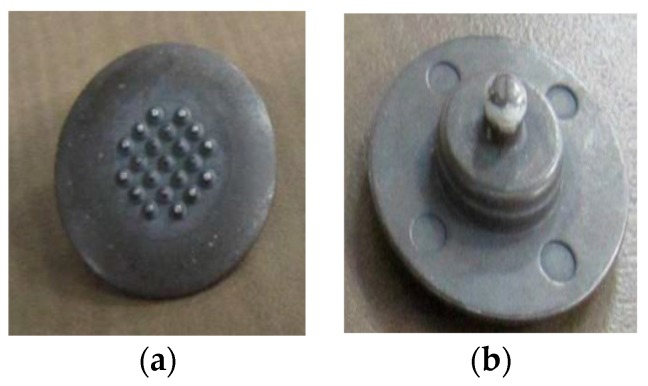
(**a**) Orbital electrode (electrode’s kin side); (**b**) Orbital electrode (electrode’s snap side).

**Figure 4:**
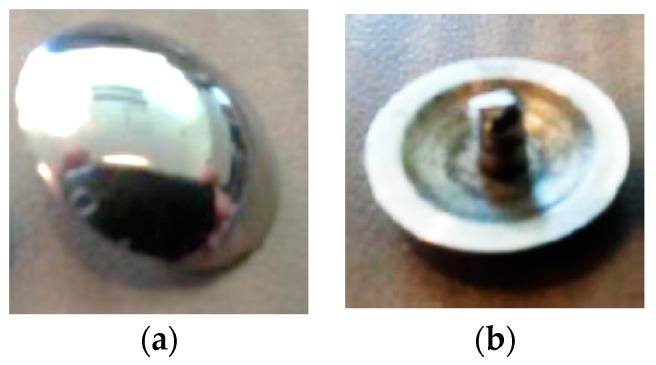
(**a**) Stainless steel electrode (electrode’s skin side); (**b**) Stainless steel electrode (electrode’s snap side).

Different surface electrode types were applied in this study. The applied electrodes used were pregelled wet surface silver/silver chloride (Ag/AgCl) electrodes (Model # FT002, MVAP II, Medical Supplies Inc., Newbury Park, CA, USA); that have a diameter of 1 cm (Figure 2). Both dry surface Orbital electrodes (Model # ORI F6T, Orbital Research Inc., Cleveland, OH, USA), which have a an effective diameter of 1.6 cm and pins (spikes) of a 150 μm length (Figure 3) and dry surface stainless steel (ST) electrodes (Model # EL12, Liberating Technologies, Inc. (LTI)**,** Holliston, MA, USA) which have a diameter of 1.42 cm and a height of 0.32 cm were applied (Figure 4). An adhesive tape was attached to Orbital and Stainless Steel electrodes to be firmly attached to the skin. Ag/AgCl electrodes had an adhesive tape by the manufacture. 

### 2.4. Equivalent Circuit Model for the Electrode-Skin Impedance 

**Figure 5:**
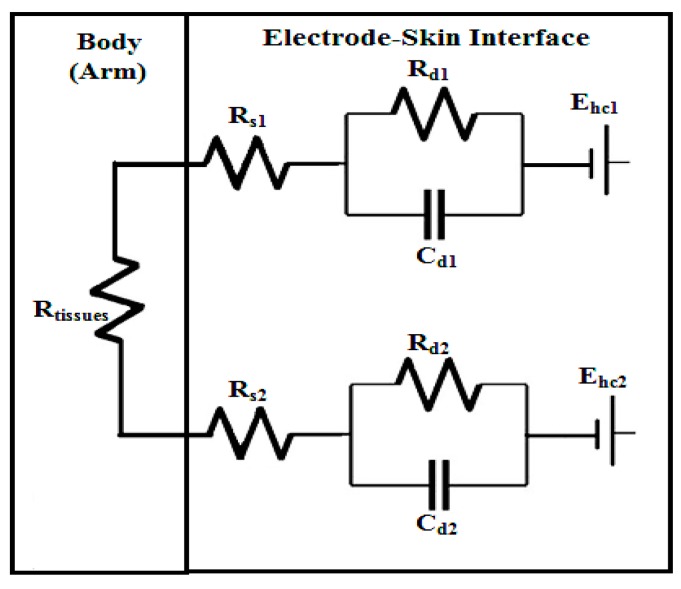
The simplified schematic diagram for the electrodes system used in the study.

The bioimpedance measurements were performed by applying two electrodes on the ventral side of the right forearm spaced 7 cm apart. The simplified schematic diagram for the electrodes system used in the study is presented in Figure 5.

In order to determine the impedance for a single electrode from two electrodes used in the study, the total impedance value is divided by two [19,22]. This approach is considered reasonable if the two electrodes are the same (e.g., identical size, identical material, produced from the same manufacture). The electrode circuit components values for the first electrode are assumed to be identical with the second electrode (C_d_ = C_d1_ = C_d2_, R_d_ = R_d1_ = R_d2_, and R_s_ = R_s1_ = R_s2_). The half-cell potential (E_hc_) represents the potential difference between the skin or electrolyte (gel or sweat) and the electrode as a result of the ions that reside between the electrode and skin [25]. The capacitance that accommodates the charges that are located between the electrode and skin double layer is represented by C_d_ [25]. The resistance that may occur to the charges transfer between the skin and electrode is represented by R_d_ [22]. The series resistance (R_s_) represents the resistance of the electrolyte gel and sweat [22]. 

The tissues resistance to the applied current is represented by R_tissues_. R_tissues_ value is generally small relative to the impedance value of the electrode-skin interface. The impedance value for healthy human arm’s tissue is found to be less than 500 Ω [9]; in contrast the impedance value for electrode-skin interface can be larger than 1 MΩ [21]. Thus, in this study, R_tissues_ is assumed to be negligible (i.e., R_tissues_ = 0). When estimating R_s_ values, any contributions from R_tissues_ are included in the R_s_ estimate. 

**Figure 6:**
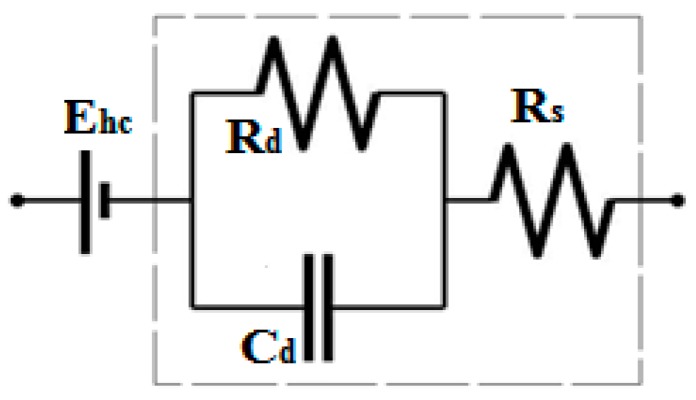
Equivalent circuit model for electrode-skin interface.

The following formula (1) is the impedance for electrode-skin interface for a single electrode. Figure 6 is a result of a simplification of the circuit of Figure 5.

(1) $$Z_{e}=R_{s}+\frac{\text{Rd}}{1+j2\pi \text{f Cd Rd}}$$

where f is the frequency (Hz).

In this study, a least squares nonlinear curve fitting method is applied using MATLAB (MATLAB version 7.7, R2008b, MathWorks Inc., Natick, MA, USA, 2008) to estimate the electrode circuit model components (R_d_, C_d_ and R_s_) values. The electrode circuit model components will be determined based on Bode plot that represents impedance as a function of frequency for electrode-skin interface [1]. Least squares nonlinear curve fitting determines the optimized best fit for impedance model based on Bode plot, in terms of total square difference from the measured impedance values. 

## 3. Results and Discussion

**Figure 7:**
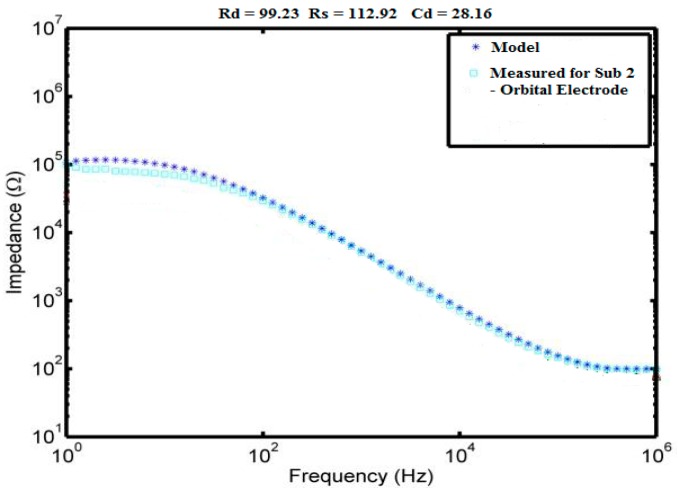
Experimental results for Orbital electrode-skin impedance frequency response (Subject, Orbital-S2) and the model plot. Estimated electrode circuit components values are located at the top of the Figure.

The estimated average values for the electrode circuit model components (R_d_, C_d_, and R_s_) for Ag/AgCl, Orbital and Stainless Steel electrodes are available in Table 2, Table 3 and Table 4 respectively. The electrode circuit model components values were estimated by applying least mean squares curve fitting method using MATLAB program. The estimated electrode circuit model values for subject 2 using orbital electrode is presented in Figure 7 as an exemplary Bode plot.

**Figure 8:**
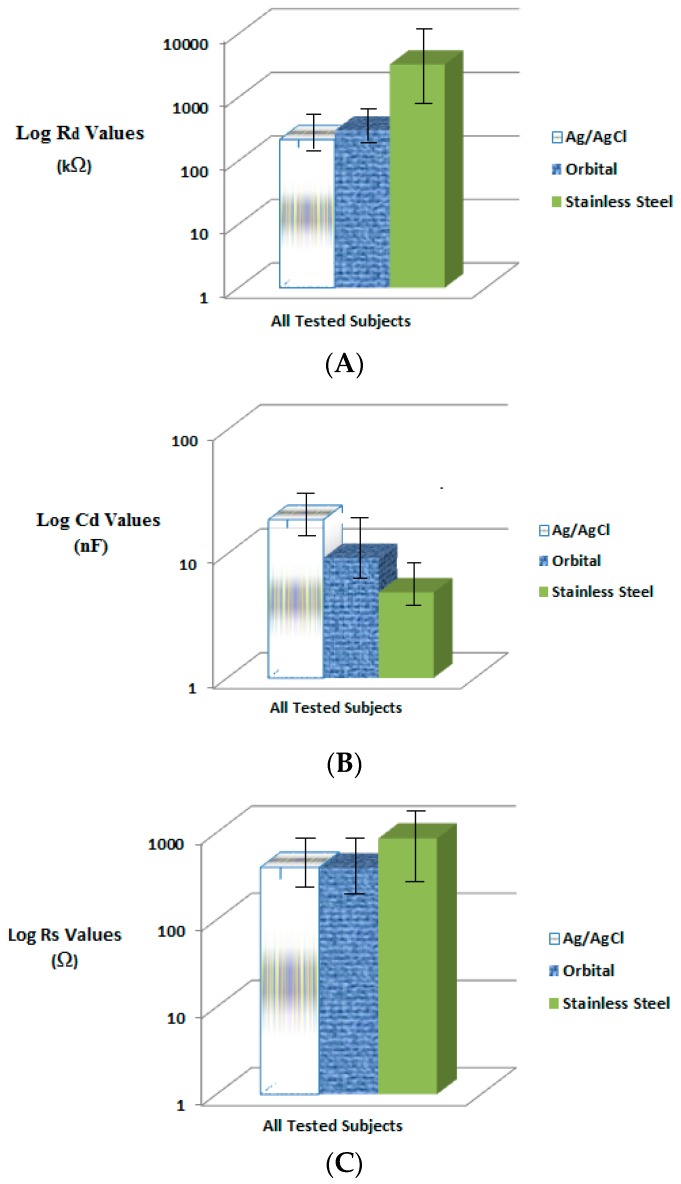
Pregelled Ag/AgCl, Orbital and Stainless Steel electrode’s circuit model components average Log values with standard deviation for all the tested subjects; (**A**) R_d_; (**B**) C_d_ and (**C**) R_s_.

The main trend for R_d_ values of Ag/AgCl electrodes is lower values in comparison to Orbital or Stainless Steel electrodes. High R_d_ value implies that the electrode-skin impedance is high. High biological signal quality requires low R_d_ value; hence choosing the type of electrode that competes with other types in having a low R_d_ value is desirable for medical devices industry. The value of resistance to ionic current that occur in the body for the biological signal can determine the quality of the signal being recorded [14,17,26]. The existence of gel at the Ag/AgCl electrodes would produce low R_d_ and R_s_ values. The existence of pins or spikes on Orbital electrodes would support the strong attachment of electrodes to skin and overcome the effect of highly resistant skin layer (stratum corneum). Low R_d_ values were obtained for Orbital electrodes that are lower than stainless steel electrodes but a bit higher than Ag/AgCl electrodes (Table 2, Table 3 and Table 4 and Figure 8A). The materials that the Orbital electrodes are made from are considered more durable than Ag/AgCl electrodes [21,26]. Therefore, Orbital electrodes can last for a longer period of time. 

The differences in electrodes’ areas were considered in reporting the electrode circuit model components values (R_d_, C_d_ and R_s_) for the three tested electrodes as reported in Table 2, Table 3 and Table 4. R_d_ mean value (215.82 kΩ/cm^2^) of Ag/AgCl electrodes is somewhat close to R_d_ mean value of Orbital electrodes (187.13 kΩ/cm^2^) with respect to surface area. However, it is much smaller than the R_d_ mean value (2130.98 kΩ/cm^2^) of Stainless Steel electrodes. 

The differences in R_d_ values of the same type of electrode among subjects are due to the difference of skin type of subjects (dry or oily), sweat secretion level and concentration of skin’s hair at electrodes sites. 

Recording biological signals at high C_d_ values is translated to better biological signal quality [1]. The measurements made by Ag/AgCl electrodes resulted in having higher C_d_ values in comparison to Orbital or Stainless Steel electrodes (Table 3 and Figure 7B). Orbital electrodes had reported high C_d_ values. The measured C_d_ values for Stainless Steel electrodes are far lower than Ag/AgCl or Orbital electrodes due to the nature of polarizable electrodes in accumulating charges at the electrode-skin sites. 

Recording biological signals at low R_s_ values is translated to better biological signal quality [5]. The existence of gel at the Ag/AgCl electrodes generated low R_s_ values (Table 4 and Figure 8C) [27]. In addition, the existence of pins or spikes in Orbital electrodes and the formation of sweat generated low R_s_ values that were close to Ag/AgCl electrodes’ R_s_ values. High R_s_ values for stainless steel electrodes resulted from the absence of electrolyte gel and were related to sweat formation. 

## 4. Conclusions

It can be concluded that pregelled Ag/AgCl electrodes would perform better than Orbital or stainless steel electrodes. Applying Ag/AgCl electrodes had resulted in having the lowest R_d_ or electrode-skin impedance values with a mean value of 215.82 (kΩ). However, Orbital electrodes that have pins in their structures had helped in generating compatible low electrode-skin impedance R_d_ values with a mean value of 299.4 (kΩ). Applying Stainless Steel electrodes resulted in having the highest R_d_ values with a mean value of 3289.4 (kΩ). Ag/AgCl electrodes had obtained the highest capacitance value (C_d_) followed by Orbital electrodes and Stainless Steel electrodes. The effect of differences in electrodes’ surface areas was considered. The existence of pins in Orbital electrodes and the formation of sweat generated low R_s_ values that were close to Ag/AgCl electrodes’ R_s_ values despite the existence of electrolyte gel on Ag/AgCl electrodes. This can be due to the existence of pins that would assist in eliminating the skin’s hair effect and strengthen the attachment to the skin. Stainless steel electrodes had resulted in high R_s_ values due to differences in material and shape. Due to the deterioration material of Ag/AgCl electrodes with time as a result of interaction with sweat, Orbital electrodes would be the most appropriate electrodes in our opinion for long time use for monitoring biological signals at home. 

## Figures and Tables

**Table 1:** Information of subjects participated in the study.

Subject	Height (cm)	Weight (kg)	Age	Gender
1	163	68	25	Male
2	174	78	28	Male
3	172	80	29	Male
4	168	65	29	Male
5	170	65	27	Male

**Table 2:** Ag/AgCl, Orbital and stainless steel electrodes’ circuit component R_d_ average values (kΩ) for all the tested subjects.

Electrode Type	Mean Values (kΩ)	kΩ/cm^2^
Ag/AgCl	215.82	215.82
Orbital	299.4	187.13
Stainless Steel	3289.4	2130.98

**Table 3:** Ag/AgCl, Orbital and stainless steel electrodes’ circuit component C_d_ average values (nF) for all the tested subjects.

Electrode Type	Mean Values (nF)	kΩ/cm^2^
Ag/AgCl	18.9	18.9
Orbital	9.3	5.2
Stainless Steel	4.9	3.45

**Table 4:** Ag/AgCl, Orbital and stainless steel electrodes’ circuit component R_s_ average values (Ω) for all the tested subjects.

Electrode Type	Mean Values (Ω)	Ω/cm^2^
Ag/AgCl	399.7	399.7
Orbital	626.8	391.8
Stainless Steel	856.4	121.1
